# Time-Delay Identification Using Multiscale Ordinal Quantifiers

**DOI:** 10.3390/e23080969

**Published:** 2021-07-27

**Authors:** Miguel C. Soriano, Luciano Zunino

**Affiliations:** 1 Instituto de Física Interdisciplinar y Sistemas Complejos (IFISC, UIB-CSIC), Campus Universitat de les Illes Balears, E-07122 Palma de Mallorca, Spain; miguel@ifisc.uib-csic.es; 2 Centro de Investigaciones Ópticas (CONICET La Plata-CIC), C.C. 3, 1897 Gonnet, Argentina; 3 Departamento de Ciencias Básicas, Facultad de Ingeniería, Universidad Nacional de La Plata (UNLP), 1900 La Plata, Argentina

**Keywords:** time-delay, time series, symbolic analysis, ordinal patterns, permutation entropy, weighted permutation entropy, Ordinal Temporal Asymmetry, autocorrelation function, linear models, nonlinear models

## Abstract

Time-delayed interactions naturally appear in a multitude of real-world systems due to the finite propagation speed of physical quantities. Often, the time scales of the interactions are unknown to an external observer and need to be inferred from time series of observed data. We explore, in this work, the properties of several ordinal-based quantifiers for the identification of time-delays from time series. To that end, we generate artificial time series of stochastic and deterministic time-delay models. We find that the presence of a nonlinearity in the generating model has consequences for the distribution of ordinal patterns and, consequently, on the delay-identification qualities of the quantifiers. Here, we put forward a novel ordinal-based quantifier that is particularly sensitive to nonlinearities in the generating model and compare it with previously-defined quantifiers. We conclude from our analysis on artificially generated data that the proper identification of the presence of a time-delay and its precise value from time series benefits from the complementary use of ordinal-based quantifiers and the standard autocorrelation function. We further validate these tools with a practical example on real-world data originating from the North Atlantic Oscillation weather phenomenon.

## 1. Introduction

Delay phenomena are commonly found in complex systems. This is essentially due to the unavoidable finite propagation speed of the physical quantities that govern the interaction between the components of these systems. Prominent examples of delay-induced complex dynamics appear in optics and life sciences [1,2].

Particularly, delayed dynamics play a key role in optical chaotic systems [3,4,5]. Secure communication at high rates with messages embedded in chaotic carriers represents one of the main practical applications within this field [6]. But what is more important for our present concerns is the fact that the success of this optical chaos-based cryptosystems strongly depends on the ability to conceal the time-delay signature, and much effort has been focused on designing new schemes able to hide it [7,8,9]. Consequently, tools specially developed to unveil this supposedly hidden information are crucial to realize how effective the concealment strategies can actually be [10].

It is also well-known that the presence of a time-delayed regulatory feedback is ubiquitous in physiological systems. Examples include respiratory, cardiac and neurological dynamics [11] (and references therein). The delay can induce chaos that increases the uncertainty, making practically impossible the long-term prediction of the system. It is clear that this *delay-induced uncertainty* can explain the difficulties observed when trying to understand the fluctuations of a measured variable related to these chaotic dynamics. As an example, we can mention the erratic evolution of the glucose level in a paradigmatic glucose–insulin model [11].

As one can easily conclude from the above description, the identification of the existence of a time-delay from experimental data is undoubtedly essential for understanding, modelling and forecasting purposes, and it deserves special consideration within the time series analysis community. Moreover, several heterogeneous scientific fields could benefit from advances along this research line. Even though several methods have been introduced for time-delay estimation from time series [12] (and references therein) [10,13], the general question about which is the optimal approach or strategy remains open. Trying to give a step forward in this direction, in this paper, we analyze the ability of three quantifiers based on ordinal patterns to unveil delay dynamics. Two of them are not new and have been implemented previously for different goals. Namely, they are the permutation entropy (PE) [14] and the weighted permutation entropy (WPE) [15]. Indeed, PE has been previously demonstrated to be particularly useful to identify the presence of delayed dynamics in nonlinear time series [16,17]. WPE improves PE in some circumstances by taking amplitude information into account. As it will be shown later in this paper, WPE helps to mitigate some of the drawbacks observed for the PE when it is used for estimating the underlying time-delay. The third ordinal tool is a novel descriptor for time-delay estimation introduced in the present work and coined as Ordinal Temporal Asymmetry here. Advantages and weaknesses associated with this new quantifier are explored to characterize its performance. Results for the widely applied autocorrelation function (ACF) have also been included as a traditional reference for time-delay recovery [18]. Comparing the behavior of these different approaches in several linear and nonlinear numerical models, relevant conclusions are obtained to deal with the delay estimation from time series in a much more efficient way. We consider that these guidelines offer a reliable strategy to practitioners for addressing the challenging task of delay identification. Finally, the presence of a delayed feedback in the daily North Atlantic Oscillation data is carefully analyzed with the aforementioned battery of tools. This real application is included to illustrate the utility of the concluded strategy in a practical setting.

## 2. Time Delay Identification Methods

### 2.1. Ordinal Symbolization Recipe

In this work, we focus on ordinal quantifiers to identify the potential presence of a time-delay from the analysis of a time series associated with an observable quantity of the dynamical system under study. The ordinal symbolic representation is obtained from the original sequence of observations by considering the relative temporal ordering of the data values. Consequently, amplitude threshold dependencies, that negatively affect other symbolization schemes, are naturally circumvented with the ordinal encoding procedure [14]. Briefly, given a one-dimensional time series, $X=\{x_{t},t=1,\ldots ,N\}$, the ordinal symbolization first requires the selection of two parameters: the order of the permutation symbols *D* (D≥2 with D∈N, length of the ordinal pattern) and the lag τ (τ∈N, time separation between time series elements). Next, the time series is mapped into subsets of length *D* of consecutive (τ=1) or non-consecutive (τ>1) values similarly to phase space reconstruction by means of time-delay-embedding. The elements in each new partition (of length *D*) are replaced by their ranks in the subset: the smallest value with 1 and the largest one with *D*. This symbolic ordering reflects the visual pattern in this subset.

**Figure 1:**
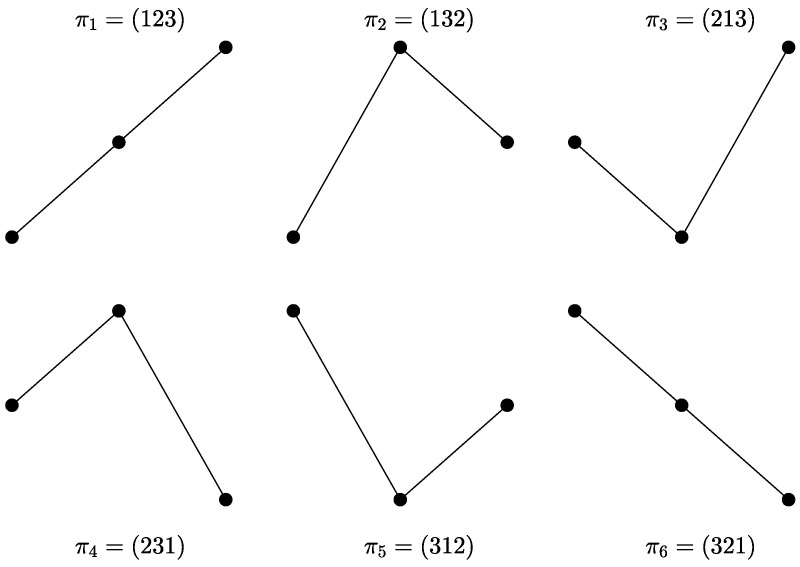
The six possible outcomes for ordinal patterns with order D=3. The amplitude of the sequence (vertical axis) is plotted as a function of the time (horizontal axis).

Figure 1 shows the 3!=6 possible ordinal patterns for D=3. With the aim of illustrating the ordinal encoding scheme, we will symbolize the short time series Y={4,1,6,5,10,7,2,8,9,3}. If D=3 and τ=1, there are eight three-dimensional vectors of three consecutive data points. The first one $\left(x_{0},x_{1},x_{2}\right)=\left(4,1,6\right)$ is mapped to the ordinal pattern $\pi_{3}=\left(213\right)$. The second three-dimensional vector is $\left(x_{1},x_{2},x_{3}\right)=\left(1,6,5\right)$, and $\pi_{2}=\left(132\right)$ will be its related permutation. The procedure continues so on until the last sequence, (8,9,3), is mapped to its corresponding motif, $\pi_{4}=\left(231\right)$. The symbolized sequence $Y_{S}$ associated with *Y* is given by $Y_{S}=\left\{\pi_{3},\pi_{2},\pi_{3},\pi_{2},\pi_{6},\pi_{3},\pi_{1},\pi_{4}\right\}$. Equal values, if any, are usually ranked according to their temporal order or, alternatively, a small random perturbation is added to break equalities [14,19,20].

The probability of each ordinal pattern can be then estimated by simply computing the relative frequencies of the D! possible permutations $\pi_{i}$:

(1) $$p\left(\pi_{i}\right)=\frac{C(\pi_{i})}{N-(D-1)\tau },i=1,\ldots ,D!$$

with $C(\pi_{i})$ the number of occurrences of the ordinal pattern $\pi_{i}$. In such a way, the ordinal pattern probability distribution, $P=\{p\left(\pi_{i}\right),i=1,\ldots ,D!\}$, is obtained. Returning to our toy numerical example: $p\left(\pi_{1}\right)=p\left(123\right)=1/8$, $p\left(\pi_{2}\right)=p\left(132\right)=1/4$, $p\left(\pi_{3}\right)=p\left(213\right)=3/8$, $p\left(\pi_{4}\right)=p\left(231\right)=1/8$, $p\left(\pi_{5}\right)=p\left(312\right)=0$, and $p\left(\pi_{6}\right)=p\left(321\right)=1/8$. For this synthetic example, as $p(\pi_{5})=0$, the ordinal pattern $\pi_{5}$ is an unobserved or forbidden ordinal pattern of the time series *Y*. When there is no temporal dependence between the values in the time series, all possible ordinal patterns appear with the same probability, i.e., $P=\{p\left(\pi_{i}\right)=1/D!,i=1,\ldots ,D!\}$. Nontrivial dynamics manifest themselves in a non-uniform distribution of the ordinal patterns. Some motifs appear more often than others and this can be interpreted as an underlying dynamical signature: the signal is less random and more predictable. This allows one to unveil hidden temporal information that helps to achieve a better understanding of the underlying mechanisms that govern the dynamics. Particularly, it is worth mentioning here that the identification of true forbidden ordinal patterns can be used to detect determinism in complex time series [21]. The ordinal symbolic mapping does not require the optimal reconstruction of the phase space that is necessary for estimating other quantifiers of chaotic signals. Consequently, *D* and τ are not usually selected following the methodologies often employed in a conventional phase space reconstruction (e.g., the first zero of the ACF, the first minimum of the average mutual information, and the false nearest neighbor algorithm) [22]. On the one hand, taking into account that there are D! potential permutations for a *D*—dimensional vector, the condition N≫D!, with *N* the length of the time series, must be satisfied in order to obtain a reliable estimation of P. It is clear that more temporal information is incorporated into the ordinal patterns as the order *D* increases. On the other hand, since the lag τ physically corresponds to multiples of the sampling time of the signal under analysis, a multiscale analysis can be easily accomplished by analyzing the behavior of any statistic of P as a function of this parameter.

### 2.2. Ordinal Entropic Quantifiers

Within the symbolic ordinal analysis, PE, just defined as the Shannon entropy of the ordinal pattern probability distribution, $S\left[P\right]=-\sum_{i=1}^{D!}p\left(\pi_{i}\right)\log\left(p\left(\pi_{i}\right)\right)$, is the most representative and widely used descriptor (0log(0) is set to zero in accordance with its mathematical limit). The PE quantifies the *temporal structural diversity* of a time series. The maximum value, $S_{\max}=\log\left(D!\right)$, is obtained for a totally random stochastic process (white noise) while the minimum value, $S_{\min}=0$, is reached for a completely regular (monotonically increasing or decreasing) time series. Regarding the time-delay identification from a time series, the behavior described by the normalized PE as a function of the lag τ, for a chosen order *D*, has been previously proposed to address this challenging task [16,17]. For irregular time series generated by delay-based systems, PE reaches a minimum when the lag τ of the symbolic reconstruction coincides with the intrinsic time-delay $\tau_{0}$ of the system, making then possible its estimation. Several practical applications have confirmed the suitability of this approach [23,24,25,26].

Amplitude information is not taken into account when the PE is estimated. That is, only the rank-order of the relative amplitudes is considered and the actual values of the data points are disregarded. Since this can be a limitation in some circumstances, different generalizations were proposed to deal with this drawback. In particular, the WPE was introduced some years ago as a modification of PE that incorporates information about the magnitude of changes between data values in addition to their relative order [15]. Being more precise, the ordinal pattern probabilities are redefined by weighting each sequence according to its variance. Hence, sequences for which the amplitude differences are greater than others have a larger contribution to the final estimated entropy value. Improved robustness for noisy signals and better characterization of data having abrupt changes in magnitude (spiky signals) are achieved with this weighted variant. For further details about the implementation of the WPE, the interested reader is referred to the seminal paper [15].

### 2.3. An Ordinal-Patterns-Based New Approach to Time-Delay Identification

When estimating standard ordinal quantifiers, the information is usually condensed into a single number that characterizes some global aspect of the underlying ordinal distribution. This could entail the loss of important details of the aforementioned distribution. Consequently, the ordinal pattern probability distribution *per se* or, alternatively, the relative frequencies of some appropriately combined ordinal patterns can result which are, for particular purposes, even more useful than PE or WPE. This hypothesis has been supported by recent studies of Bandt [27,28], Cuesta-Frau et al. [29] and Gunther et al. [30]. Actually, it has been previously shown that hierarchies and probabilities of the ordinal patterns offer a better characterization of the dynamical regimes of some complex systems, identifying transitions and behaviors that are not detected by more traditional statistical tools [31,32,33,34]. Ordinal patterns analysis can also be helpful to forecast extreme events in complex dynamics such as those obtained from an optically injected semiconductor laser [35], a semiconductor laser with optical feedback in the low frequency fluctuations regime [36], and a Raman fiber laser in the chaotic regime [37]. Besides, it is worth highlighting that the predictive capability achieved with ordinal patterns can go beyond the one reached by more conventional tools as it has been very recently shown for the complex dynamics of the paradigmatic Duffing oscillator [30]. Finally, probabilities of time-lagged ordinal patterns, i.e., ordinal patterns estimated with non-consecutive data points (τ≥2), provide suitable features for efficient classification of biomedical signals allowing to improve the discriminative power of well-established biomarkers [38].

**Figure 2:**
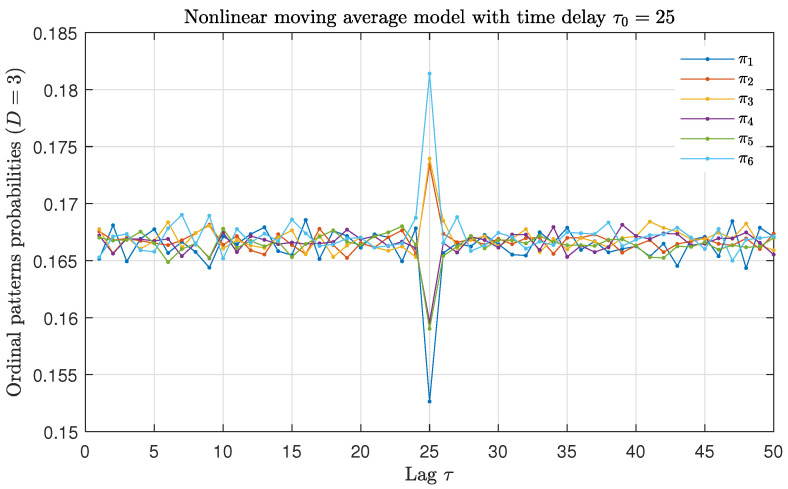
Ordinal patterns probabilities with D=3 as a function of the lag τ for the nonlinear moving average model with feedback strength α=0.1 and intrinsic time-delay $\tau_{0}=25$. Results obtained for a representative realization of length $N=10^{5}$ data are plotted.

All these previous findings make us wonder if the analysis of a suitable combination of all or some of the ordinal motifs could not be more revealing than global ordinal descriptors, such as PE or WPE, regarding the time-delay identification purpose. Trying to give a step forward in this direction, we have analyzed how the probability of the six ordinal patterns with order D=3 changes as a function of the lag τ for numerical realizations of length $N=10^{5}$ data of the nonlinear moving average model with feedback strength α=0.1 and time-delay $\tau_{0}=25$. More details on this delayed stochastic model will be included in the next section, see Equation (6). Results obtained for a representative realization are displayed in Figure 2. A clear hierarchy is observed when the lag τ matches the intrinsic time-delay $\tau_{0}=25$ of the model: ordinal patterns $\pi_{6}$, $\pi_{2}$ and $\pi_{3}$ have larger probabilities than those estimated for their time reversal ordinal counterparts $\pi_{1}$, $\pi_{4}$ and $\pi_{5}$, respectively. We have verified a similar behavior for the same model with other values of α and $\tau_{0}$, and also for other stochastic and chaotic models with delay. That is, we heuristically observe that the absolute values of the difference between the probabilities of the time reversal pairwise ordinal patterns: $\left|p\left(\pi_{1}\right)-p\left(\pi_{6}\right)\right|$, $\left|p\left(\pi_{2}\right)-p\left(\pi_{4}\right)\right|$ and $\left|p\left(\pi_{3}\right)-p\left(\pi_{5}\right)\right|$ are maximized when the lag τ coincides with the delay $\tau_{0}$ of the system. The absolute value should be included since according to the nature of the system (linear/nonlinear, stochastic/chaotic) the ordinal pattern of the time reversal pair with the larger probability changes. Taking into account that the main aim of the present work is the identification of the intrinsic time-delay of the system, we define the Ordinal Temporal Asymmetry (OTA) as:

(2) $$\mathrm{OTA}=\left|p\left(\pi_{1}\right)-p\left(\pi_{6}\right)\right|+\left|p\left(\pi_{2}\right)-p\left(\pi_{4}\right)\right|+\left|p\left(\pi_{3}\right)-p\left(\pi_{5}\right)\right|.$$

It is expected that this quantifier reaches a clear maximum when $\tau =\tau_{0}$. Thus, we argue that the time-delay of the system can be estimated by analyzing how the OTA behaves as a function of the lag τ and looking for the aforementioned maximum. It is worth noticing here that the same previously introduced ordinal pattern pairs have been used by Zanin et al. [39] to propose a reversibility statistical test: a time series is reversible if and only if ordinal patterns composing the previous pairs have approximately the same probability. This allows us to conjecture that the irreversibility degree of the system is maximized at the intrinsic time-delay scale.

The OTA function, whose definition is based on the ordinal patterns with D=3 (Equation (2)), can be directly generalized for larger orders (D>3) as the sum of the absolute values of the difference between the probabilities of the time reversal pairwise ordinal patterns for these orders *D*. An alternative practical way of doing this is to calculate the half of the sum of the absolute values of the difference between the probabilities of the ordinal patterns of the original ($p_{orig}\left(\pi_{i}\right)$) and time-reversed ($p_{rev}\left(\pi_{i}\right)$) time series:

(3) $$\mathrm{OTA}\left(D\right)=\frac{1}{2}\sum_{i=1}^{D!}\left|p_{orig}\left(\pi_{i}\right)-p_{rev}\left(\pi_{i}\right)\right|.$$

This generalized measure quantifies the amount of irreversibility of the time series and, consequently, it is expected that the OTA, so defined, reaches a maximum at the time-delay scale, where the nonlinearity is maximized. Moreover, since larger orders yield a richer description of the underlying dynamics, an improved identification of the time-delay could be achieved by using D>3. In the next sections, we characterize strengths and weaknesses of the OTA as a tool to unveil the presence of a (hidden) time-delay in comparison to other related techniques.

### 2.4. Autocorrelation Function

Here, we also consider the normalized ACF, which is a standard statistical method very often used to identify the time-delay from time series [18]. We consider it as a reference to which compare the performance of the ordinal quantifiers (PE, WPE and OTA). Given the time series $X=\{x_{t},t=1,\ldots ,N\}$, the autocorrelation for lag τ measures the correlation between $x_{t}$ and $x_{t+\tau }$ as

(4) $$r_{\tau }=\frac{c_{\tau }}{c_{0}},$$

where

(5) $$c_{\tau }=\frac{1}{N}\sum_{t=1}^{N-\tau }\left(x_{t}-\langle X\rangle \right)\left(x_{t+\tau }-\langle X\rangle \right)$$

with 〈X〉 the sample mean of the time series and $c_{0}$ a normalization factor such that $r_{0}=1$. The value $r_{\tau }$ is also called sample autocorrelation function by Box et al. [40] (page 31).

## 3. Numerical Analysis

In this section, we apply the above-mentioned quantifiers to time series that originate from numerical simulations of different linear and nonlinear models with time-delay. We pay particular attention at how the different quantifiers react to the presence of a time-delay in the generating model. To that end, we evaluate the quantifiers described in Section 2 for multiples values of the lag. When analyzing the different time series, we expect to observe an abrupt change in the quantifiers when the lag matches the time-delay of the underlying models if the identification is successful.

### 3.1. Stochastic Models

#### 3.1.1. Moving Average Models

We start by analyzing the time series generated by a nonlinear moving average (NLMA) model with time-delay. The model is defined as follows:

(6) $$X_{t}=\alpha \epsilon_{t-\tau_{0}}^{2}+\epsilon_{t},$$

where $\epsilon_{t}$ is a sequence of independent and identically normally distributed random variables with mean equal to 0 and variance equal to 1, α is the feedback strength and $\tau_{0}$ is the intrinsic time-delay.

**Figure 3:**
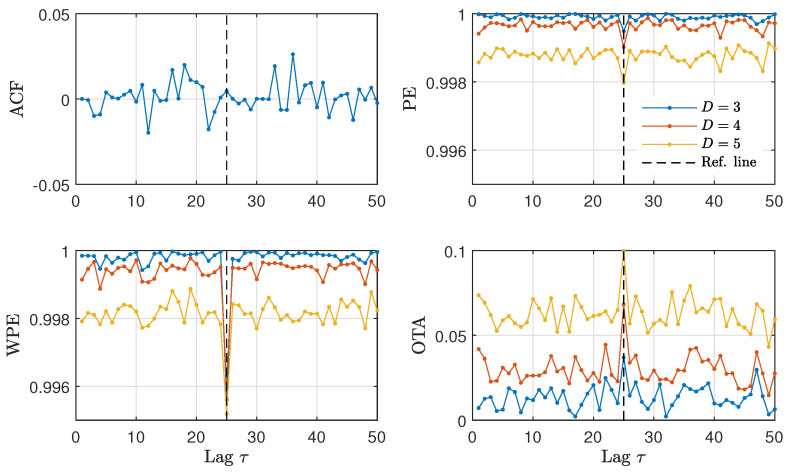
ACF and ordinal quantifiers estimated with several orders *D* as a function of the lag τ (1≤τ≤50) for the NLMA model with feedback strength α=0.1 and intrinsic time-delay $\tau_{0}=25$. Results obtained for a representative realization of length $N=10^{4}$ data are plotted.

Figure 3 shows the numerical results for the estimation of ACF, PE, WPE and OTA at different values of the lag for time series generated by the nonlinear stochastic model presented in Equation (6), with parameters α=0.1 and $\tau_{0}=25$. We find that the ACF fluctuates around zero, without any noticeable change in its value when $\tau \approx \tau_{0}$, denoted as a dashed vertical line in Figure 3. In contrast, the ordinal quantifiers show an abrupt change when $\tau \approx \tau_{0}$. Both the PE and the WPE identify the presence of a time-delay in the analyzed time series, showing a downward peak for all orders *D*. The OTA identifies the presence of a time-delay as well, in this case with an upward peak.

**Figure 4:**
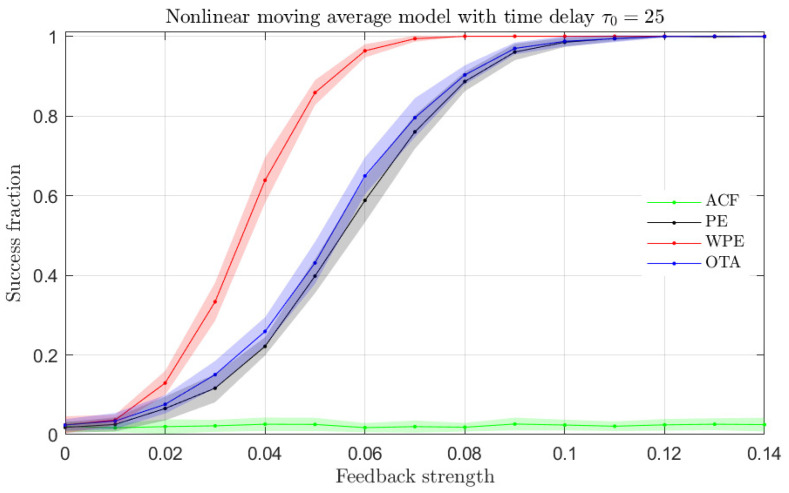
Fraction of success identification of the time-delay for the NLMA model with intrinsic time-delay $\tau_{0}=25$ as a function of the feedback strength for the ACF and ordinal quantifiers estimated with order D=4 (qualitatively similar behaviors are obtained for orders D=3 and D=5). One hundred independent realizations of length $N=10^{4}$ data were tested to calculate the success fraction. Mean (colored solid lines) and standard deviation (colored shaded regions) from twenty success fraction estimations (one hundred realizations each) are plotted.

From the results presented in Figure 3, it becomes apparent that the ordinal quantifiers are more sensitive than the ACF to the presence of a time-delay in the NLMA time series. In order to validate this finding, we present in Figure 4 the fraction of successful time-delay identifications as a function of the feedback strength α in Equation (6) for all quantifiers. A successful time-delay identification refers to the presence of an upward or downward peak in the quantifiers, distinct to the respective quantifier values for other lags. We find that the ordinal quantifiers start to distinguish the presence of a time-delay for α≳0.01 from the analyzed time series, while the ACF fails to identify the presence of a time-delay regardless the value of the feedback strength. Among the ordinal quantifiers, the WPE is more successful than the PE and the OTA for feedback strengths in the range 0.01≲α≲0.1.

As shown here, the ACF fails to identify the presence of a time-delay in NLMA time series. In line with the arguments put forward by Faura et al. [13], we argue that the nonlinearity in the model is the main factor that reduces the efficacy of the ACF.

In order to keep contrasting the different properties of the quantifiers, we next proceed to analyze time series generated by a linear moving average model. A linear moving average (LMA) model can be defined as follows:

(7) $$X_{t}=\alpha \epsilon_{t-\tau_{0}}+\epsilon_{t}$$

with $\epsilon_{t}$, α, $\tau_{0}$ defined as in Equation (6).

**Figure 5:**
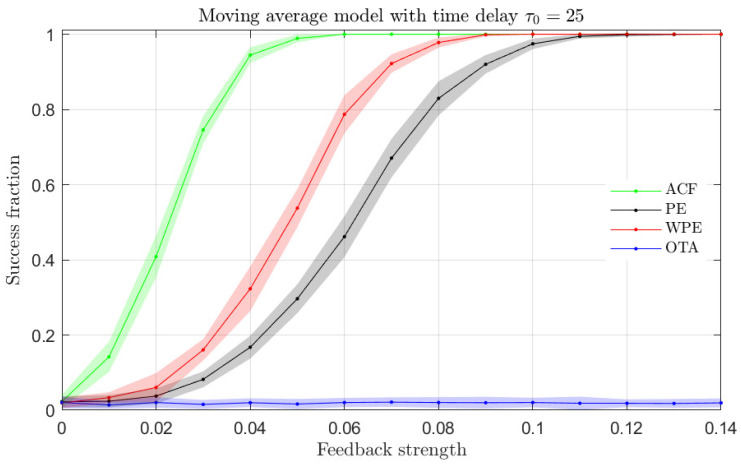
Same as Figure 4 but for the LMA model.

We generate time series from the linear stochastic model described in Equation (7) with $\tau_{0}=25$ and varying feedback strength α. Following the procedure introduced above for Figure 4, we now compute the estimation of ACF, PE, WPE and OTA for the numerically generated LMA time series and evaluate the fraction of successful time-delay identifications for all these quantifiers. As shown in Figure 5, we find that the ACF, PE and WPE quantifiers can all successfully identify the presence of a time-delay in the time series.

In the case of the results for the LMA model presented in Figure 5, the ACF is the most sensitive quantifier to the presence of a time-delay, followed by the WPE and the PE. The OTA quantifier fails to identify the time-delay of the underlying model from the analyzed time series. We note that the OTA quantifier has been introduced to detect asymmetries in the temporal ordinal patterns of the analyzed time series, which are not present in time series originating from linear systems as illustrated here.

In summary, the only quantifiers that are able to distinguish the presence of a time-delay for both LMA and NLMA models are the WPE and the PE. In the analyzed time series of the NLMA and LMA models, the WPE has a larger fraction of successful time-delay identifications than the PE. The observation that the OTA is only sensitive to the delay in the NLMA model and the ACF is only sensitive to the delay in the LMA model will be further investigated in the next section with other types of stochastic models, namely Gaussian and non-Gaussian auto-regressive models.

#### 3.1.2. Auto-Regressive Models

We continue by analyzing the time series generated by a Gaussian auto-regressive (AR) model with time-delay. The model is defined as follows:

(8) $$X_{t}=\alpha X_{t-\tau_{0}}+\epsilon_{t},$$

where $\epsilon_{t}$, α, $\tau_{0}$ are defined as in Equation (6).

**Figure 6:**
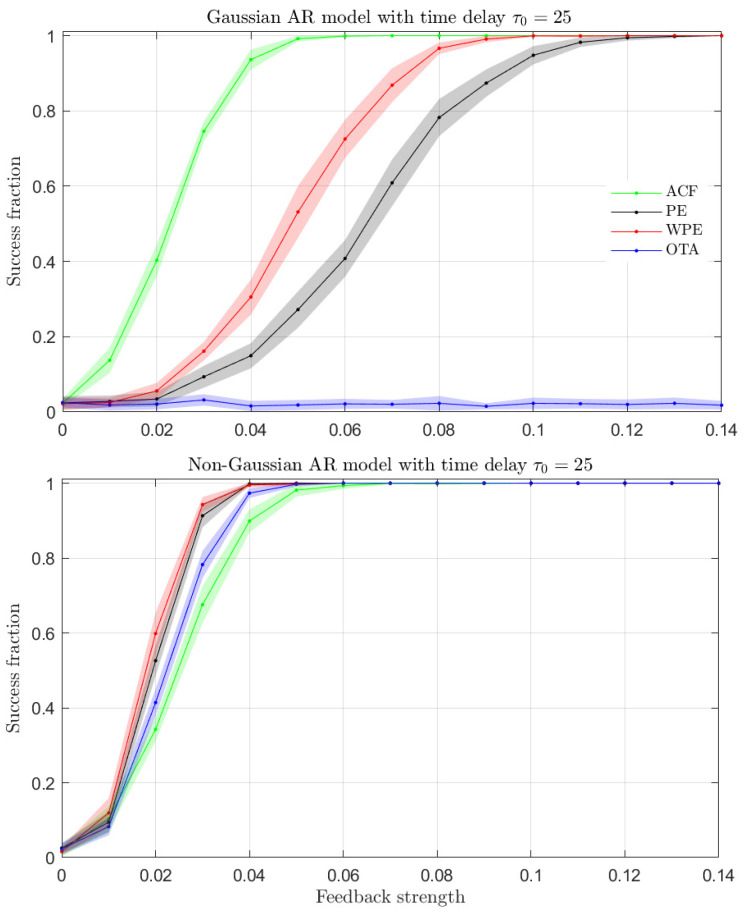
Same as Figure 4 but for the Gaussian (**top**) and non-Gaussian (**bottom**) AR models.

Figure 6 (top) shows the fraction of successful time-delay identifications as a function of the feedback strength α for the Gaussian AR time series with the ACF, PE, WPE and OTA quantifiers. We find that the ACF quantifier is the most sensitive to the presence of a time-delay in the time series, with the highest success ratio for low and intermediate feedback strengths. From the ordinal quantifiers, the WPE is more successful than the PE for the time-delay identification, while the OTA is unable to identify a time-delay in the analyzed time series. The results for the time series generated by the Gaussian AR model are qualitatively similar to the results for the LMA model presented in Figure 5. In particular, the OTA does not detect the presence of a time-delay in the analyzed linear models.

One can break the linearity of the generating AR model by considering a noise with a non-Gaussian (log-normal) distribution. The non-Gaussian AR model with time-delay is defined as follows:

(9) $$X_{t}=\alpha X_{t-\tau_{0}}+\mu_{t}$$

with α the feedback strength and $\tau_{0}$ the intrinsic time-delay as before while $\mu_{t}$ is now a sequence of independent and identically log-normally distributed random variables with mean equal to 0 and variance equal to 1.

Figure 6 (bottom) shows the fraction of successful time-delay identifications for the non-Gaussian AR time series with the ACF, PE, WPE and OTA quantifiers. We find that all quantifiers successfully identify the time-delay present in the time series with a similar accuracy. In this case, the WPE is the most successful quantifier, followed closely by the PE, OTA and ACF quantifiers.

The results presented in Figure 6 illustrate that the OTA is able to identify the presence of a time-delay if the generating model has a nonlinearity, here originated from the log-normal distribution of the noise. The presence of a nonlinearity breaks the temporal symmetry of the system and the OTA can then identify the delay of the generating model. For both the Gaussian and non-Gaussian AR models, the WPE is the most sensitive ordinal quantifier. Finally, the ACF is able to identify the delay even for the non-Gaussian AR model, showing that it is not restricted to linear stochastic models.

We have also confirmed qualitatively similar findings in the case of an ARMA model, where the AR and MA components are mixed with two different delays: ACF, PE and WPE estimate both of them appropriately while the OTA is not able to unveil their presence. On the other hand, when NLMA and AR are mixed with two different delays, it is observed that the ACF is only able to unveil the delay related to the linear dynamics whereas OTA is only sensitive to the delay associated with the nonlinear counterpart. PE and WPE detect both of them, but the identification is not as clear as that realized by ACF and OTA. So, the combination of ACF and OTA seems to be the optimal recipe to deal with this challenging situation.

### 3.2. Deterministic Chaotic Models

Nonlinear systems with time-delay can produce high-dimensional deterministic chaos [41,42]. Such systems have received extensive attention from the scientific community due to their long-term unpredictability even though they are generated in a deterministic manner [43,44]. Here, we focus on the analysis of time series generated by the time-delayed Hénon map, defined as follows:

(10) $$X_{t}=1-aX_{t-1}^{2}+bX_{t-\tau_{0}},$$

where $\tau_{0}$ is the intrinsic time-delay. The values of the parameters *a* and *b* have been chosen equal to 1.6 and 0.1, respectively. Chaotic solutions are obtained in such a case for all $\tau_{0}\geq 1$ [45,46].

**Figure 7:**
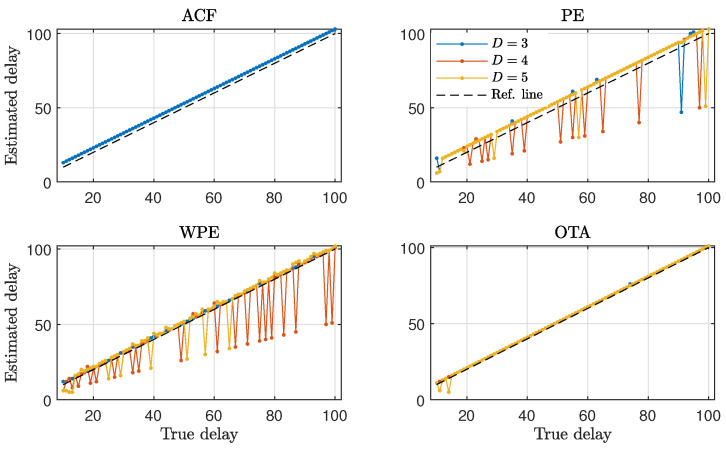
Estimated time-delay as a function of the true time-delay for the time-delayed Hénon map with $10\leq \tau_{0}\leq 100$. Performance of the ACF and ordinal quantifiers with several orders *D* are contrasted. Results obtained for representative realizations of length $N=10^{4}$ data are plotted.

We first explore the capability of the ACF, WPE, PE and OTA quantifiers to identify the presence of a time-delay in the time series generated by the time-delayed Hénon map of Equation (10). In nonlinear systems with time-delay, it is often of importance to estimate the precise value of the time-delay from the time series [7,10]. Thus, we present in Figure 7 the estimated delay for all the analyzed quantifiers as a function of the true time-delay, $\tau_{0}$, of the generating chaotic model.

Figure 7 shows that the ACF can properly identify the presence of a time-delay in the generated time series in the range $10\leq \tau_{0}\leq 100$ of analyzed delays. However, we find that the ACF systematically overestimates the value of the time-delay, which is evidenced by the offset between the estimated time-delay (blue solid line in Figure 7) and the true time-delay (dashed line in Figure 7). The PE and WPE ordinal quantifiers, as shown in Figure 7, can also identify the presence of the time-delay in the generated time series. These two ordinal quantifiers exhibit a certain tendency to estimate the value of the time-delay at approximately half the true time-delay values for orders D>3. This delay misidentification happens when the value of the quantifier for lags around the sub-multiple of the true time-delay gives a higher contrast than the value of the quantifier for lags around the true time-delay. The presence of delay sub-multiples in the analysis of time series is a known feature of the PE and its variants [17]. In addition, the time-delay values estimated by the PE have an offset with respect to the true time-delays, while the delays estimated by the WPE deviate less from the true time-delays. Finally, the OTA quantifier can properly identify the presence of a time-delay. Values of the time-delay estimated by the OTA deviate little from the true time-delay values, as indicated by the overlap between the solid (estimated delay) and the dashed (true delay) lines in Figure 7.

**Figure 8:**
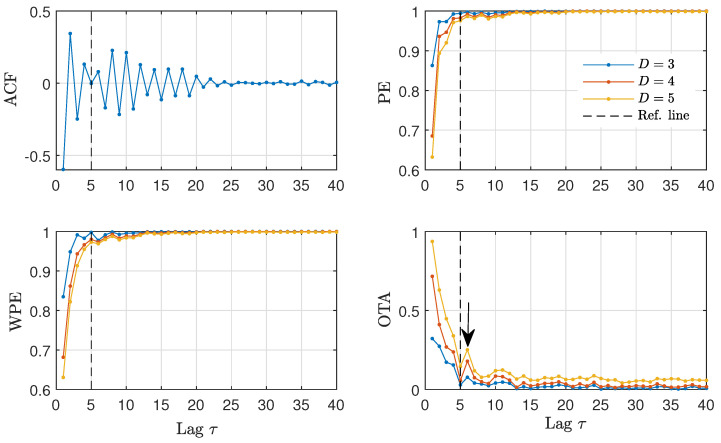
ACF and ordinal quantifiers estimated with several orders *D* as a function of the lag τ (1≤τ≤40) for the time-delayed Hénon map with intrinsic time-delay $\tau_{0}=5$. The arrow indicates a maximum for the OTA at τ=6. Results obtained for a representative realization of length $N=10^{4}$ data are plotted.

The identification of time-delays from time series becomes more intricate when the values of the time-delay are in the vicinity of other temporal scales present in the generating models [47]. For this reason, we proceed to analyze time series from the time-delayed Hénon map with intrinsic time-delay $\tau_{0}=5$. As shown in Figure 8, the ACF, WPE and PE quantifiers hardly identify the presence of a time-delay, indicated by the dashed vertical line in this figure. The OTA quantifier is the only one that has a clear change for lags around the true time-delay, indicated by the arrow in Figure 8.

The suitability of the OTA quantifier for the identification of time-delays in time series originated by the time-delayed Hénon map is highlighted by the results presented in Figure 7 and Figure 8. We have checked that the favorable properties of the OTA quantifier for time-delay identification are similarly observed when analyzing other deterministic chaotic models such as the delayed logistic map.

## 4. An Illustrative Real Application

In the previous section, we have evaluated the battery of ordinal-based quantifiers on artificial data. Here, we put them to test on the analysis of real world data with recordings that originate from the so-called North Atlantic Oscillation [48]. More precisely, daily data from 1 January 1950 to 30 November 2020 (N=25,900) have been analyzed. These data are freely available at the Climate Prediction Center website (https://www.cpc.ncep.noaa.gov/ (accessed on 15 February 2021)). The presence of a delayed feedback in the North Atlantic Oscillation data has been questioned in the literature, with a characteristic time scale in the order of several days [49,50,51]. We have identified this problem as being a suitable practical example to test the ACF, PE, WPE, and OTA quantifiers since different methods appear to give different values for such characteristic feedback time.

**Figure 9:**
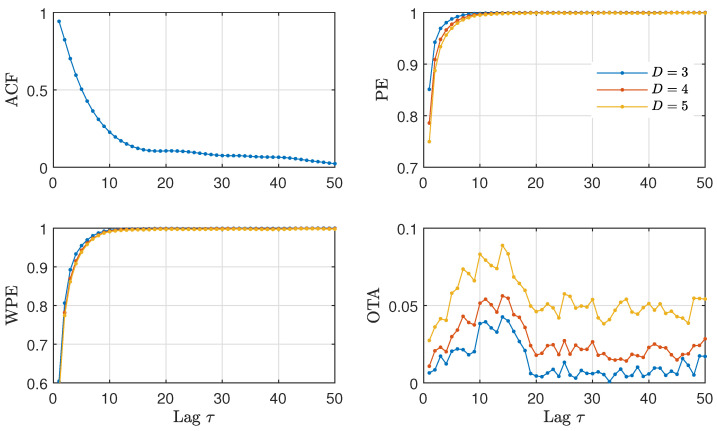
ACF and ordinal quantifiers estimated with several orders *D* as a function of the lag τ (1≤τ≤50) for the daily North Atlantic Oscillation data (N=25,900). A maximum of the OTA for a lag τ between 10 and 15 days is observed for the three orders (D=3, D=4 and D=5).

In Figure 9, we analyze the time series of the daily North Atlantic Oscillation data in order to check the existence of a delayed feedback and, if possible, to quantify its characteristic feedback time. We find that the ACF decays almost monotonically, with a *shoulder* around 20 days. The PE and WPE quantifiers grow monotonically with no apparent change that could qualify as a time-delay. In contrast, the OTA exhibits a clear maximum around a lag of 10 to 15 days. These estimated values for the feedback time are in agreement with the autocorrelation analysis performed in Ref. [49]. Here, we show that the characteristic time appears more clearly with the OTA as compared to the ACF. According to the literature on the topic, this characteristic feedback time could correspond to intraseasonal time-scales that arise due to stratosphere-troposphere interactions [49]. For this data, a separation between winter and summer appears to be relevant for a more precise geophysical interpretation of the time scales [51], which is however beyond the scope of this paper.

An interesting property of the OTA for delays that are relatively short or delays that are close with respect to other time scales of the system becomes apparent in Figure 9. The OTA has a low value for most lags, with the peak at the delay rising from the background. The high contrast of the OTA in this setting of short delays is not present in the ACF, with the delay appearing as a shoulder over a decaying envelope, or in the WPE/PE, with a small change over a rising envelope.

The results shown in Section 3 and Section 4 illustrate that the OTA offers properties that complement previously-developed tools and may be useful to practitioners for addressing the challenging task of time-delay identification from irregular time series.

## 5. Conclusions

Here, we have explored, in detail, the properties of several multiscale ordinal quantifiers for the identification of time-delay systems from time series. We contrasted the information extracted by such ordinal quantifiers with the standard ACF. By analyzing the outcomes of the different quantifiers on linear and nonlinear model systems with time-delay, we have observed some general trends than can be summarized as follows. First, we have shown that the standard ACF captures the presence of a time-delay for all linear models and some nonlinear ones. As illustrated by the example of the NLMA model, the ACF can fail to identify the presence of a time-delay in some nonlinear models. Second, the PE succeeds in finding the time-delay in all the analyzed artificial models. Some potential problems that the PE is subject to are the precise identification of the time-delay value in systems with inertia and delay misidentifications due to the presence of spurious time-delay sub-multiples. Third, the WPE inherits good properties of the PE and partly mitigates its problems with the inertia and the sub-multiples. Finally, the OTA identifies the presence of the time-delay in all nonlinear models and can eventually fail for a linear one, as illustrated by the LMA and Gaussian AR models. We have observed that the OTA is more precise in the identification of the time-delay value than the other quantifiers in the case of the deterministic chaotic models with inertia. An additional advantage of the OTA is its ability to detect small delays, which we have illustrated with the practical application in the North Atlantic Oscillation data.

Our results highlight the complementarity of the multiscale ordinal quantifiers with standard ACF, as well as the practical need of analyzing time series that originate from time-delay systems with battery of tools that are as diverse as possible.

## Data Availability

The data presented in this study are available on request from the corresponding author.

## Abbreviations

The following abbreviations are used in this manuscript:

ACF	Autocorrelation Function
AR	Auto-regressive
LMA	Linear Moving Average
NLMA	Nonlinear Moving Average
OTA	Ordinal Temporal Asymmetry
PE	Permutation Entropy
WPE	Weighted Permutation Entropy
